# *De Novo* Emergence of Peptides That Confer Antibiotic Resistance

**DOI:** 10.1128/mBio.00837-19

**Published:** 2019-06-04

**Authors:** Michael Knopp, Jonina S. Gudmundsdottir, Tobias Nilsson, Finja König, Omar Warsi, Fredrika Rajer, Pia Ädelroth, Dan I. Andersson

**Affiliations:** a Department of Medical Biochemistry and Microbiology, Biomedical Center, Uppsala University, Uppsala, Sweden; b Department of Biochemistry and Biophysics, Stockholm University, Stockholm, Sweden; McMaster University; Centro Nacional de Biotecnología CSIC; San Diego State University

**Keywords:** *Escherichia coli*, antibiotic resistance, aminoglycosides, *de novo*, gene evolution, membrane potential, peptides

## Abstract

The origin of novel genes and beneficial functions is of fundamental interest in evolutionary biology. New genes can originate from different mechanisms, including horizontal gene transfer, duplication-divergence, and *de novo* from noncoding DNA sequences. Comparative genomics has generated strong evidence for *de novo* emergence of genes in various organisms, but experimental demonstration of this process has been limited to localized randomization in preexisting structural scaffolds. This bypasses the basic requirement of *de novo* gene emergence, i.e., lack of an ancestral gene. We constructed highly diverse plasmid libraries encoding randomly generated open reading frames and expressed them in Escherichia coli to identify short peptides that could confer a beneficial and selectable phenotype *in vivo* (in a living cell). Selections on antibiotic-containing agar plates resulted in the identification of three peptides that increased aminoglycoside resistance up to 48-fold. Combining genetic and functional analyses, we show that the peptides are highly hydrophobic, and by inserting into the membrane, they reduce membrane potential, decrease aminoglycoside uptake, and thereby confer high-level resistance. This study demonstrates that randomized DNA sequences can encode peptides that confer selective benefits and illustrates how expression of random sequences could spark the origination of new genes. In addition, our results also show that this question can be addressed experimentally by expression of highly diverse sequence libraries and subsequent selection for specific functions, such as resistance to toxic compounds, the ability to rescue auxotrophic/temperature-sensitive mutants, and growth on normally nonused carbon sources, allowing the exploration of many different phenotypes.

## Abstract (precis)

*De novo* gene origination from nonfunctional DNA sequences was long assumed to be implausible. However, recent studies have shown that large fractions of genomic noncoding DNA are transcribed and translated, potentially generating new genes. Experimental validation of this process so far has been limited to comparative genomics, *in vitro* selections, or partial randomizations. Here, we describe selection of novel peptides *in vivo* using fully random synthetic expression libraries. The peptides confer aminoglycoside resistance by inserting into the bacterial membrane and thereby partly reducing membrane potential and decreasing drug uptake. Our results show that beneficial peptides can be selected from random sequence pools *in vivo* and support the idea that expression of noncoding sequences could spark the origination of new genes.

## INTRODUCTION

Different mechanisms for the acquisition of new genes, such as horizontal gene transfer and duplication-divergence, have been observed in both natural systems and experimental settings (1, 2). However, these mechanisms rely on preexisting genes as a substrate for evolution and do not explain how these preexisting genes arose *de novo* from noncoding DNA (3, 4). For a protein-coding gene to arise *de novo*, a noncoding nucleotide sequence has to be transcribed and translated and confer a selectable beneficial function. The evolution of an entirely new gene without any functional progenitor has been regarded as an exceedingly rare and improbable event (5), and considering that a random sequence generated by chance must encode a stable polypeptide with a function accessible by selection, this notion of impossibility might seem reasonable. However, comparative genomics in eukaryotes such as insects (6), yeast (7), Hydra (8), primates (9, 10), mouse (11, 12), *Plasmodium* (13), and plants (14) has provided strong evidence that this is possible and that parts of extant genomes contain genes without any recognized homologous sequences that nature had “tinkered” with.

In addition, it is becoming clear that large portions of the genomes of many organisms, including noncoding sequences, are highly transcribed (15, 16), and that these transcripts can contain randomly occurring small open reading frames (sORFs) that are translated (17–19). Historically, sORFs have been largely overlooked due to missing annotations and their incompatibility with many molecular biology detection tools. An increasing number of studies employing comparative genomics and ribosome profiling, however, show that sORFs are pervasive among all investigated organisms and can make up a large fraction of prokaryotic genomes (19–22). Modern tools for gene prediction that incorporate ribosome profiling data estimate the presence of 125 sORFs encoding proteins of ≤71 amino acids in Escherichia coli (22). Another study based on sequence conservation and ribosome binding site models predicts 217 sORFs encoding peptides of less than 50 amino acids, most of which contain predicted transmembrane domains (23). This pervasive expression of small proteins potentially can serve as a pool for the *de novo* selection of functional peptides encoded by sORFs (20, 21, 23). Furthermore, small proteins have been shown to carry out versatile functions, including attenuation of gene expression and regulation of transporters, stress response, and signal transduction (23–26). The increasing recognition of sORFs as an abundant and functionally diverse class of genes that could originate from stochastic transcription and translation of noncoding sequences make them well suited for studying their *de novo* emergence by experimental evolution. In this study, we examined if short peptides encoded by randomized DNA sequences that were expressed from a plasmid in E. coli cells could confer selective benefits in the form of antibiotic resistance and by which mechanisms such new phenotypes could be generated.

## RESULTS AND DISCUSSION

### Library construction and screening for *de novo* sORFs.

**FIG 1:**
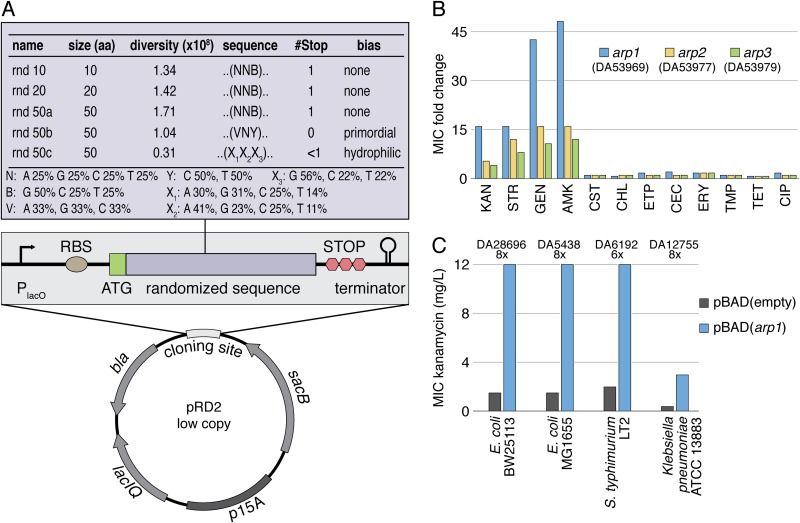
Selection of peptides from randomized sequence libraries that increase antibiotic resistance. (A) Expression vector and sequence libraries. Randomized sequences were cloned in the low-copy-number expression vector pRD2 with flanking transcriptional and translational start and stop sequences. The five libraries encode peptides comprised of 10 to 50 amino acids, with a total diversity of 5.82 × 10^8^. Repeats of NNB encode random amino acid compositions, while VNY repeats are biased toward primordial amino acids (Ala, Asp, Glu, Gly, Ile, Leu, Pro, Ser, Thr, and Val) and X_1_X_2_X_3_ repeats enrich for hydrophilicity to promote intrinsic disorder and functional promiscuity. (B) Cross-resistance of Arp1, Arp2, and Arp3 to different antibiotics: kanamycin (KAN), streptomycin (STR), gentamicin (GEN), amikacin (AMK), colistin (CST), chloramphenicol (CHL), ertapenem (ETP), cefaclor (CEC), erythromycin (ERY), trimethoprim (TMP), tetracycline (TET), and ciprofloxacin (CIP). A complete strain list, including custom identifiers (DA numbers), can be found in Table S1. Absolute MIC values are listed in Table S2. (C) Functionality of Arp1 in different bacterial strains and species. DA number and fold increase are indicated above the graph.

We constructed a set of highly diverse plasmid libraries containing randomly generated sORFs (Fig. 1A). The plasmid used is an in-house construct with a low-copy-number origin of replication (p15A). The synthetic sORFs are under the control of an inducible P*_LlacO_* promoter and encode peptides ranging from 10 to 50 amino acids. To avoid premature stop codons, we excluded adenine from the third codon position. This resulted in a 3.9-fold increase in full-length 50-mers compared to NNN (N = A, C, G, and T) repeats (see Fig. S1A in the supplemental material) while still encoding all amino acids with only mildly changed frequencies (Fig. S1B). Two libraries were designed with biased nucleotide compositions encoding either a higher fraction of primordial amino acids (27) (rnd_50b) or hydrophilic amino acids to increase intrinsic disorder and promote functional promiscuity (28) (rnd_50c) (Fig. S1C). The diversity of each library was between 0.3 × 10^8^ and 1.7 × 10^8^, which is the typical upper limit of transformation efficiency. However, it is noteworthy that these libraries only cover a marginal fraction of the complete possible diversity, which is approximately 9.5 × 10^33^ for a random 50-mer protein. All five libraries were transformed into Escherichia coli K-12 strain BW25113 (here designated E. coli) and screened for ORFs that increase antibiotic resistance. For this, cells carrying each plasmid library were spread on plates containing antibiotics above the MIC of an E. coli strain carrying the empty control plasmid. In total, 5.82 × 10^8^ cells, each expressing a unique sORF, were tested on 10 different antibiotics representing 9 antibiotic classes (kanamycin, trimethoprim, tetracycline, amikacin, fosfomycin, chloramphenicol, rifampin, cefaclor, nitrofurantoin, and ciprofloxacin) at three different concentrations, ranging from 2-fold to 16-fold of the respective MIC. Selection on plates was chosen over bulk selection in liquid culture to reduce the influence of selective sweeps and also allow the isolation of variants with low fitness.

10.1128/mBio.00837-19.1 FIG S1 (A) Full-length probabilities of 50mers composed of NNB (dashed lines) or NNN repeats (straight line), where N = A, C, G, or T and B = C, G, or T. (B) Sequences composed of NNN (N = A, C, G, or T) and NNB (B = C, G, or T) repeats encode similar amino acid frequencies. (C) Repeats of VNY (V = A [33%], G [33%], or C [33%]; Y = C [50%] or T [50%]) encode only 12 amino acids, 10 of which are so-called prebiotic amino acids. Repeats of X_1_X_2_X_3_ (X_1_ = A [30%], G [31%], C [25%], or T [14%]; X_2_ = A [41%], G [23%], C [25%], or T [11%]; X_13_ = G [56%], C [22%], or T [22%]) encode all amino acids with a strong bias towards hydrophilicity. Download FIG S1, PDF file, 1.0 MB. Copyright © 2019 Knopp et al. 2019 Knopp et al. This content is distributed under the terms of the Creative Commons Attribution 4.0 International license.

10.1128/mBio.00837-19.6 TABLE S1 List of strains used in this study. Download Table S1, PDF file, 0.1 MB. Copyright © 2019 Knopp et al. 2019 Knopp et al. This content is distributed under the terms of the Creative Commons Attribution 4.0 International license.

10.1128/mBio.00837-19.7 TABLE S2 MICS of different antibiotics for strains carrying an empty vector control or expressing Arp1-3. Values represent the concentration, in mg/liter, of the respective antibiotic that inhibited growth. Download Table S2, PDF file, 0.1 MB. Copyright © 2019 Knopp et al. 2019 Knopp et al. This content is distributed under the terms of the Creative Commons Attribution 4.0 International license.

### Isolation of peptides increasing aminoglycoside resistance.

We isolated three unique sORFs (designated Arp, for aminoglycoside resistance peptide) on plates containing kanamycin. Two of these sORFs were also selected on amikacin plates. To ensure that the observed phenotypes were caused by expression of the sORFs rather than by unintended resistance mutations in the chromosome, we recloned all inserts into an empty plasmid and confirmed the phenotypes in the absence and presence of the inducer isopropyl-β-d-thiogalactopyranoside (IPTG). Furthermore, we showed that the function was dependent on the encoded protein rather than the RNA transcript by demonstrating that variants without start codons or introduced frameshift mutations lost activity, and that inserts maintained activity when the identical amino acid sequence was recoded with a different nucleotide sequence (Table S3). To determine the resistance levels provided by Arp1 to Arp3 (Arp1-3) as well as their ability to confer cross-resistance to other antibiotics, we measured the MICs of 12 different antibiotics representing the major classes (Fig. 1B and Fig. S2). All peptides increased the MICs of the aminoglycosides kanamycin, amikacin, gentamicin, and streptomycin. Arp1 had the strongest effect, increasing amikacin resistance 48-fold compared to that of a strain with an empty control vector. The susceptibility to other antibiotic classes was not altered by overexpression of any of the peptides, demonstrating that the effect was specific to one antibiotic class. We also tested the dependence of the phenotype on the expression vector, copy number, P*_LlacO_* promoter, and host background. Overexpression of Arp1 caused a similar increase in resistance when expressed from a single chromosomal copy under the control of P*_LlacO_* or when placed under an arabinose-inducible promoter on a medium-copy-number plasmid (pBAD18 backbone [29]). Increased resistance was also observed when Arp1 was overexpressed in the E. coli K-12 strain MG1655, Salmonella enterica Typhimurium LT2, and Klebsiella pneumoniae ATCC 13883 (Fig. 1C), showing that the mechanism of action is independent of the expression system and the specific bacterial strain/species.

10.1128/mBio.00837-19.2 FIG S2 **Expression of Arp1-3 causes heterogeneity in resistance levels in the populations.** (A to D) Overexpression of Arp1-3 using the P*_LlacO_*-based expression system in pRD2 induces the formation of subpopulations with various minimal inhibitory concentrations (MICs). Arp1 causes the largest fraction of cells that tolerate 6 mg/liter kanamycin. (E and F) Using the arabinose-inducible expression system in pBAD18 further increases the fraction of the subpopulation with the highest resistance level. Values represent the means from 4 independent experiments. Numbers in brackets represent the standard deviations. We observed multiple zones of inhibition when performing MIC determinations using Etest strips with strains overexpressing Arp1-3. This was an indication of a heterogeneous population that prompted us to quantify the observed effect. Plating of an overnight culture on plates containing various amounts of kanamycin showed that after overexpression of Arp1 using pRD2, only 35% of the population was able to tolerate 6 mg/liter kanamycin. Subpopulations of various resistance levels were also observed after overexpression of Arp1 and Arp2. When overexpressed from pBAD18, Arp1 still induced the formation of subpopulations; however, the fraction of higher resistance cells increased to 50%. Thus, we decided to use this expression system for downstream experiments. Several causes could explain this effect: stochasticity could cause various expression levels of the peptide in different cells of the same population. Furthermore, the proposed resistance mechanism depends on insertion of the peptides into the membrane followed by a depolarization. The extent of membrane insertion could also randomly fluctuate among different cells. Lastly, it is likely that membrane potential itself shows some variation among different cells; thus, cells might be differentially affected by insertion of the same amount of peptide into the membrane. While the mechanistic basis of the formation of subpopulations is unclear, it limits our ability for phenotypic characterization. For example, the presence of populations with wild-type MIC, and therefore presumably a wild-type membrane potential, will reduce the signal we are able to measure for the membrane potential-responsive probe DiBAC_4_(3) (Fig. 3B) and the uptake of radiolabeled dihydrostreptomycin (Fig. 3C). Download FIG S2, PDF file, 0.1 MB. Copyright © 2019 Knopp et al. 2019 Knopp et al. This content is distributed under the terms of the Creative Commons Attribution 4.0 International license.

10.1128/mBio.00837-19.8 TABLE S3 Verification of hits. To ensure the phenotype was mediated by the encoded peptide, the level of resistance conferred after recoding, induction by IPTG, removal of the start codon, and introduction of a frame shift was determined. +, growth on plates containing 6 mg/liter kanamycin; (+), weak growth; −, no growth. Download Table S3, PDF file, 0.1 MB. Copyright © 2019 Knopp et al. 2019 Knopp et al. This content is distributed under the terms of the Creative Commons Attribution 4.0 International license.

### Arp1-3 are small, hydrophobic membrane peptides.

**FIG 2:**
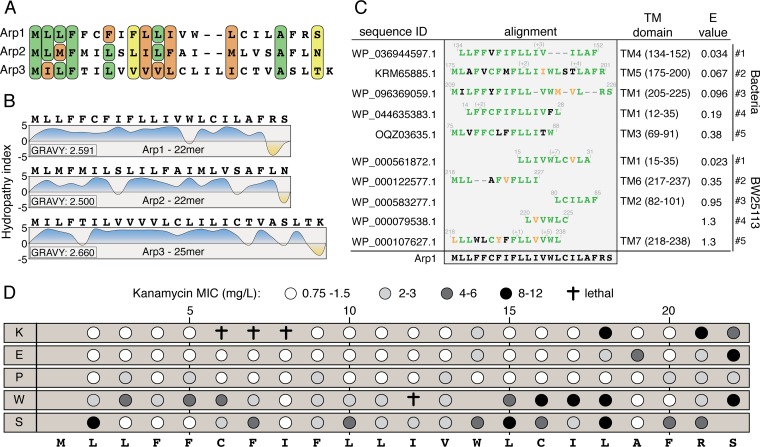
Sequence analysis of the resistance peptides. (A) Alignments of the selected peptides show sequence similarities for specific positions and peptide length. Green backgrounds indicate identical amino acids, while orange and yellow indicate strongly and weakly similar properties, respectively. (B) Hydropathy profiles (30). Averages of hydropathy scores (GRAVY) are indicated for each peptide. (C) BLAST-hit analysis of Arp1 against bacteria and E. coli K-12 BW25113. Identical amino acids are marked in green, and similar amino acids are marked in orange. (D) Site-directed mutagenesis of Arp1. The white-to-black scale indicates the level of kanamycin resistance conferred by the mutant peptide. †, the mutant peptide is toxic when expressed.

The sORFs originated from the rnd_50A (Arp1 and Arp3) and rnd_50B (Arp2) libraries, but sequence analysis revealed that all three sORFs encoded truncated peptides (due to inadvertent introduction of stop codons or frameshifts during synthesis/cloning), resulting in two 22mers (Arp1 and Arp2) and one 25mer (Arp3). Besides the similarities in length, all peptides had a high content (80 to 86%) of hydrophobic residues (Fig. 2A and B). The C terminus contained hydrophilic amino acids in all three peptides, suggesting a functional role in the observed phenotype. The very high average hydropathy (GRAVY) scores of all three peptides (≥2.5) indicate a probable localization in the membrane (30), which was also predicted by the transmembrane α-helix prediction tool TMHMM with high confidence. Additionally, a BLASTp search for Arp1 against all bacteria or only the E. coli K-12 strain BW25113 showed that 9 out of the top 10 hit sequences were located in transmembrane domains (Fig. 2C).

Since all three peptides showed strong similarities in sequence composition, namely, a highly hydrophobic sequence followed by a hydrophilic C terminus, we conducted a site-directed mutagenesis experiment based on Arp1 to identify functionally important positions (Fig. 2D). Each residue was replaced with a positively charged (lysine), negatively charged (glutamic acid), α-helix breaker (proline), bulky (tryptophan), and hydrophilic (serine) amino acid. The 103 variants generated were then analyzed for their effect on the MIC of kanamycin to test which residues in Arp1 are required for the observed resistance phenotype and which residues can be varied. Changes to charged amino acids caused a complete loss of function in most cases, except for five variants with C-terminal substitutions that maintained kanamycin MICs of over 4 mg/liter (compared to MICs of the empty vector control of 0.75 mg/liter and Arp1 of 12 mg/liter). Changes to proline resulted in 15 nonfunctional variants and 6 variants with a MIC of 2 to 3 mg/liter kanamycin. In contrast, the peptide was more tolerant to replacements with tryptophan and serine, with only 4 variants each losing complete functionality.

**FIG 3:**
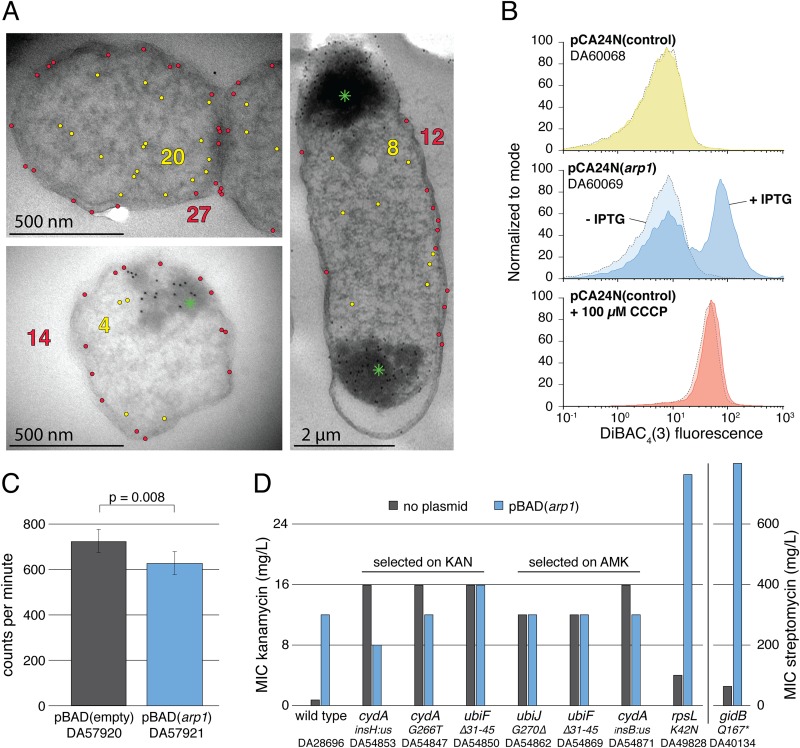
Localization and mechanism of action of Arp1. (A) Transmission electron microscopy of immunogold-labeled samples expressing His-tagged Arp1. The green asterisks indicate inclusion bodies at the cell poles. Gold particles outside the inclusion bodies are colored yellow or red if they are located in the cytoplasm or cell periphery, respectively. Original micrographs can be found in Fig. S5A. (B) Membrane potential determined by the slow-response-potential-sensitive probe DiBAC_4_(3). The protonophore CCCP was used as a positive control causing a complete collapse of membrane potential. Shown is a representative data set from three independent assays (Fig. S5B). (C) Uptake of radiolabeled dihydrostreptomycin in cells carrying a control plasmid or overexpressing Arp1. Bars are the means from five biological replicates, and error bars represent standard deviations. (D) Additivity of the peptide-mediated resistance phenotype with various mutants of E. coli and *S.* Typhimurium LT2. The relevant genetic changes of the six chromosomal mutants are indicated below. A strain list, including custom identifiers (DA numbers) and complete genotypes of all mutants, can be found in Tables S1 and S4.

The strong sensitivity to charged amino acids and to the α-helix breaker proline further supports the predicted transmembrane α-helix (31) and that the membrane insertion is important for the increase in resistance. To show the membrane localization of the identified peptides, we fused a hexahistidine tag to the C terminus of Arp1 and conducted an electron microscopic analysis of immunogold-labeled samples using a His tag antibody (Fig. 3A). The micrographs revealed large inclusion bodies in the cells, a common artifact when overexpressing hydrophobic peptides (32, 33), which are strongly decorated with antibody-attached gold particles. Outside these aggregates the antibodies localized both in the cytoplasm as well as in the cell periphery, indicating that a major fraction of free Arp1 (outside the inclusion bodies) does populate the membrane. We observed strong heterogeneity of gold particle distribution among different cells, which indicates stochastic differences of expression and stability of Arp1 among different cells. Additionally, it is reasonable to assume that since the phenotype was selected in a single step, it is imperfect compared to most genes that have evolved for long times to conduct specific functions.

10.1128/mBio.00837-19.5 FIG S5 (A) Transmission electron microscopy. E. coli MG1655 expressing His-tagged Arp1 was immunogold labelled and visualized using electron microscopy. The position of Arp1 is marked by black dots. (B) Membrane potential assay. The membrane potential was determined by the slow-response-potential-sensitive probe DiBAC_4_(3). The protonophore CCCP was used as a positive control causing a complete collapse of membrane potential. Shown are two replicates of the data presented in Fig. 3B. Download FIG S5, PDF file, 0.2 MB. Copyright © 2019 Knopp et al. 2019 Knopp et al. This content is distributed under the terms of the Creative Commons Attribution 4.0 International license.

10.1128/mBio.00837-19.9 TABLE S4 Whole-genome sequencing results of six chromosomal mutants selected on 6 mg/liter kanamycin (DA54853, DA54847, and DA54850) or amikacin (DA54862, DA54869, and DA54871). Insertions of IS5 (*insH*), IS1 (*insB*), and adenine (A) occurred upstream (us) of the corresponding gene. Download Table S4, PDF file, 0.1 MB. Copyright © 2019 Knopp et al. 2019 Knopp et al. This content is distributed under the terms of the Creative Commons Attribution 4.0 International license.

### Resistance is mediated via reduced aminoglycoside uptake due to membrane depolarization.

How can a peptide with a short transmembrane α-helix cause an increase in resistance to a whole class of antibiotics? It is known that aminoglycoside uptake relies on the membrane potential and that resistance can be conferred by reducing the membrane potential (34, 35), and conceivably these peptides could act in a similar way. To test this idea, we determined if overexpression of Arp1 causes a disruption of the electrochemical gradient by using the membrane potential-sensitive probe bis-(1,3-dibutylbarbituric acid)trimethine oxonol [DiBAC_4_(3)] (Fig. 3B). The protonophore carbonyl cyanide m-chlorophenyl hydrazine (CCCP), which collapses the membrane potential, was used as a positive control. After CCCP treatment, a clear shift to higher fluorescence occurred due to an increased uptake of DiBAC_4_(3). This shift in fluorescence was also observed upon induction of Arp1 expression from the high-copy-number plasmid (pCA24N), showing that Arp1 insertion in the membrane causes a reduction of membrane potential. Additionally, we observed that expression of Arp1-3 results in the formation of subpopulations of cells with different resistance levels (Fig. S2), which might be due to cellular heterogeneity in expression and/or solubility of the peptides. The resulting difference in amounts of free peptides that can insert into the membrane could cause differences in membrane depolarization across the population and thereby generate cells with different resistance levels. This heterogeneity decreases the sensitivity of the conducted assays, since only a fraction of the population shows the investigated phenotype (Fig. S2). We also determined if the uptake of streptomycin was reduced when Arp1 is expressed. As shown in Fig. 3C, levels of intracellular radiolabeled dihydrostreptomycin were significantly lower in a strain expressing the Arp1 (Fig. 3C). The observed decrease in intracellular streptomycin is relatively small, although statistically significant (*P* = 0.008), but reduced streptomycin uptake and increased resistance does not necessarily correlate in a proportional way. Thus, a small effect on uptake can have a large effect on resistance, as has been observed previously (36).

To determine if overexpression of Arp1 in strains with elevated aminoglycoside resistance can cause an additional increase in resistance levels, we selected chromosomal mutants with the empty vector control strain on the same concentrations of kanamycin and amikacin that were used for selection of the *de novo* resistance genes. We isolated 24 mutants (Fig. S3) and determined the resistance level to both antibiotics. Strikingly, all mutants exhibited approximately the same level of resistance as that observed for Arp1 overexpression. Furthermore, the selected mutants exhibited cross-resistance to both kanamycin and amikacin, indicating an identical mechanism of action. Whole-genome sequencing of six clones showed that all had mutations in genes associated with electron transport and generation of membrane potential (Table S4). Overexpression of Arp1 in these mutants did not cause any further increase in kanamycin or amikacin resistance, indicating that the chromosomal mutations and peptides act by a similar mechanism (i.e., reduction in membrane potential) and that Arp1-dependent depolarization causes the highest resistance increase achievable by this mechanism. In contrast, overexpression of Arp1 in strains with mutations in the antibiotic target (*rpsL* K42N) or a target modification enzyme (*gidB* Q167*) elevated resistance levels beyond the level that was observed for mutants with disrupted membrane potentials (Fig. 3D).

10.1128/mBio.00837-19.3 FIG S3 **Selection of chromosomal mutants**. Twenty-four chromosomal mutants were selected on 6 mg/liter kanamycin or amikacin. All mutants showed cross-resistance to both antibiotics. The resistance levels were similar to the resistance level provided by overexpression of Arp1. Download FIG S3, PDF file, 0.1 MB. Copyright © 2019 Knopp et al. 2019 Knopp et al. This content is distributed under the terms of the Creative Commons Attribution 4.0 International license.

The sORFs selected from our randomized sequence libraries were shown to (i) localize in the cell membrane, (ii) disrupt membrane potential, (iii) consequently increase aminoglycoside resistance to the maximum amount that can be achieved by this resistance pathway, and (iv) show additive effects when combined with resistance mechanisms that are unrelated to the membrane potential (i.e., target modification). The results presented here are fully compatible with the depolarization model, but it should be pointed out that natural small proteins, such as MgtS (37), AcrZ (38, 39), and others (24), have been shown to regulate membrane-located transporters, including efflux pumps such as AcrAB (39). Thus, hypothetically Arp1-3 could confer increased resistance by upregulating an efflux pump. However, this explanation is unlikely, since the peptide-conferred resistance is completely specific for aminoglycosides, with no effect on more typical hydrophobic efflux pump substrates such as tetracyclines, chloramphenicol, and fluoroquinolones.

Alternatively, albeit unlikely, the increased resistance to aminoglycosides could be based on a physiological response to high expression of foreign peptides. As seen in Fig. 3A, production of Arp1 causes the formation of inclusion bodies. This will likely result in a disruption of the normal cell physiology and cause the activation of stress responses (40, 41). For example, the heat shock response induces upregulation of various chaperones and proteases that could themselves influence the formation of membrane potential. However, to our knowledge, no stress response exists in E. coli that results specifically in aminoglycoside resistance. Furthermore, if the effect was due to formation of inclusion bodies and generation of a stress response, one would expect that overexpression of many peptides would generate resistance (which we do not observe), since overexpression is often associated with formation of inclusion bodies. Finally, if the resistance was due to the reduced growth rate associated with peptide production, one would expect that unrelated mutations that reduce growth rate should induce aminoglycoside resistance as well. However, analysis of aminoglycoside resistance in several unrelated mutants with reduced growth rate had no effect on aminoglycoside resistance (42). In summary, it seems very unlikely that the aminoglycoside resistance is caused by an indirect physiological effect.

**FIG 4:**
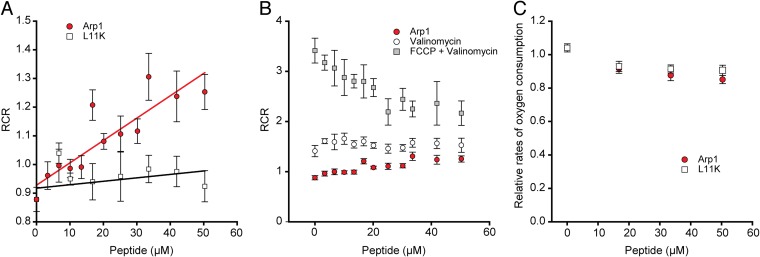
*In vitro* studies with synthesized Arp1 and Arp1-L11K peptides. (A) The steady-state activity of bovine Cyt*c*O in liposomes after addition of Arp1 (red circles) and L11K (white squares) divided by the coupled (original) rate gives the RCR. The data point at 0 μM peptide indicates the value obtained with only the solvent DMSO added. (B) RCRs for Arp1 with addition of valinomycin (6 μM, white circles) and FCCP (21 μM, gray squares) in addition to the peptide. (C) Relative oxygen consumption rates for Cyt*c*O in DDM detergent micelles with addition of either Arp1 (red circles) or L11K (white squares). All data points in panels A to C are averages with standard errors, *n* > 3.

To further examine if the loss of membrane potential is a direct effect of peptide insertion into the membrane, we used a purified *in vitro* system to measure the effect of synthesized Arp1 peptide and a nonfunctional (i.e., confers no resistance when expressed *in vivo*) control variant (Arp1-L11K) (Fig. 2D) on the activity of the proton pump bovine cytochrome *c* oxidase (Cyt*c*O) reconstituted into proteoliposomes (Fig. 4A; also see schematic in Fig. S4). When the enzyme is active, it reduces oxygen to water, using protons from inside the vesicles; in addition, protons are pumped across the membrane. A proton electrochemical gradient thereby is rapidly built up across the liposome membrane, slowing down further activity, and the resulting slow steady-state rate is called the coupled rate. When ionophores (valinomycin, a K^+^ ionophore, and/or FCCP, a protonophore similar to CCCP) are added, this gradient is partly or fully dissipated, and the so-called uncoupled rate is reached. Respiratory control ratios, dividing the uncoupled and the coupled rates (RCR; see reference 43), are a measure of the degree to which additives can uncouple the built-up potential and generally gives a value of 3 to 5 for full uncoupling by FCCP in our experimental system (see Fig. S4 for a schematic of the experiment and an example of a raw data trace). Thus, we determined the RCRs after adding the Arp-1 and Arp1-L11K peptides, and as positive controls we added valinomycin and FCCP in addition to the peptides (Fig. 4B and Fig. S4C). Increasing amounts of Arp1 had mild uncoupling effects (i.e., stimulated the activity of Cyt*c*O), with RCRs increasing up to around 1.4 (Fig. 4A and B). Valinomycin addition led to further rate increase, and FCCP fully collapsed the gradient, giving the maximum Cyt*c*O activity. The L11K variant of the peptide did not show any uncoupling effect (Fig. 4A and Fig. S4C), even at high concentrations. The maximal RCR after FCCP addition decreased at higher peptide concentrations, indicating that it affects the Cyt*c*O negatively (Fig. 4B and Fig. S4C). We further investigated if the peptides indeed affect the enzyme activity *per se* by measuring their effect on the Cyt*c*O activity in detergent. As seen in Fig. 4C, both peptides had small negative effects on the Cyt*c*O activity in detergent. Thus, the activity increase observed in the Cyt*c*O liposomes when the Arp1 peptide is added is most likely due to an uncoupling effect, as the direct effect of peptide itself on the oxidase is negative. In summary, these results show that Arp1 is capable of partly uncoupling a proton electrochemical gradient, and that the likely cause for this uncoupling is an increase in the permeability to protons. That the peptide does not completely dissipate the gradient is important, since full dissipation would stop bacterial growth.

10.1128/mBio.00837-19.4 FIG S4 **Schematic of and data from the Cyt*c*O-liposome experiments.** (A) Cyt*c*O reduces O_2_ to H_2_O, using electrons from reduced cytochrome (cyt.) *c* and protons from inside the liposomes. In addition, protons are pumped and a proton electrochemical gradient (positive outside) across the membrane is established. Valinomycin, a K^+^ ionophore, can dissipate the charge component and FCCP, a protonophore, both components of this gradient. (B) A typical oxygen consumption experiment; the baseline with only ascorbate, TMPD, and cyt. *c* was first measured. Proteoliposomes then were added, initiating oxygen consumption by Cyt*c*O and build-up of a membrane potential. The coupled rate is the slope after liposome addition subtracting the baseline. Peptides (Arp1 or L11K), valinomycin, and FCCP were added, in this order, and the rates measured. RCRs were determined by dividing the different rates by the initial coupled rate. (C) RCRs for Arp1-L11K (as shown for Arp1 in Fig. 4B) after addition of the peptide (black circles) plus valinomycin (6 μM; white circles) and FCCP (21 μM; grey squares). Data points are averages with standard errors, *n* > 3. Download FIG S4, PDF file, 0.1 MB. Copyright © 2019 Knopp et al. 2019 Knopp et al. This content is distributed under the terms of the Creative Commons Attribution 4.0 International license.

### Increased aminoglycoside resistance is specific for Arp1-3.

How easily can peptide-mediated membrane depolarization be achieved? Theoretically, any peptide or domain that spans the membrane creates a lesion in the phospholipid bilayer that could cause leakage of protons or ions. However, the site-directed mutagenesis of Arp1 (Fig. 2D) showed that the function is sequence specific. Not only did most changes to charged amino acids or prolines cause a complete loss of function but also substitutions with a weak hydrophilic residue (serine) or even another hydrophobic amino acid (tryptophan) were detrimental in almost all variants. Additionally, four variants (C6K, F7K, I8K, and I12W) were toxic when expressed. While we do not know the cause of the cytotoxicity, it is possible that the substitutions cause a lethal disruption of membrane integrity. This shows that not only functionality but also viability is quickly lost upon changes of the specific sequences that were isolated.

Furthermore, the alignment of Arp1-3 suggests that, besides the requirement of a strong hydrophobicity followed by a hydrophilic C terminus, certain positions are conserved. For example, Arp1-3 all contained a phenylalanine at position 4, which indicates a functional role of this residue possibly promoting dimerization (44). To further elucidate the sequence requirements and conservation, more variants of this peptide class need to be isolated to generate a higher statistical power which allows a more detailed analysis. To further show that the phenotype is specific to Arp1-3, we overexpressed three endogenous E. coli peptides, TisB, IbsC, and LdrD, that are similar to Arp1-3 in regard to size and hydrophobicity. The 29-mer toxin TisB, which is encoded by the E. coli K-12 genome, is part of a toxin-antitoxin system, and it inhibits growth when overexpressed. It has been shown to form channels that allow ion leakage and cause membrane depolarization (45, 46). However, TisB expression from a pBAD promoter (arabinose concentrations ranging between 0.0125 and 0.2%) conferred no increased resistance when expressed at viable levels (high expression levels of TisB were lethal). Similarly, no increased resistance could be observed when overexpressing the toxins IbsC and LdrD. We further tested if overexpression of other transmembrane domains that are not part of toxin-antitoxin systems could cause aminoglycoside resistance. For this, we chose 10 representative transmembrane domains from 5 genes with different physiological functions (Table S5) and overexpressed them from the pBAD18 vector. None of the variants increased resistance to kanamycin or amikacin. These results show that Arp1-3 are facilitating a unique function in causing depolarization to an extent that generates antibiotic resistance but still maintains cell viability.

10.1128/mBio.00837-19.10 TABLE S5 List of transmembrane helices tested for increased aminoglycoside resistance. Download Table S5, PDF file, 0.1 MB. Copyright © 2019 Knopp et al. 2019 Knopp et al. This content is distributed under the terms of the Creative Commons Attribution 4.0 International license.

### Beneficial and deleterious effects conferred by membrane depolarization.

A reduction of membrane potential is expected to be deleterious for bacteria in most environments, since the electrochemical gradient across the inner membrane promotes various physiological functions, such as ATP synthesis, nutrient flux, and pH homeostasis. However, under certain conditions, mutants with a defective electron transport chain have a strong selective advantage. For example, small-colony variants (SCVs), which often result from mutations in genes associated with electron transport, have been isolated from various infections and species (47). Additionally, SCVs can be rapidly selected *in vitro*, for example, under antibiotic pressure (48, 49), cold stress (50), hydrostatic pressure (51), or low pH (52). The reduced membrane potential and low growth rate of electron transport-defective SCVs offer various advantages to the bacterial cell. First, the uptake of toxic compounds that rely on an active membrane potential, like aminoglycoside antibiotics, is reduced and the cell will be able to persist and/or proliferate. Additionally, the reduced growth rate and, thus, reduced speed of cell division will dampen the cytostatic effect of β-lactam antibiotics (53). Besides the selective advantage in the presence of antibiotics, SCVs can have an increased survival rate *in vivo*. These mutants are often attenuated, for example, by a decreased expression of immunogens, which causes a reduction in immune response and therefore a higher survival rate inside the host (47). Other clinically important resistance mechanisms, in particular target alterations/modifications, are typically associated with a reduction in bacterial fitness in the absence of antibiotics (54). For example, mutations in *rpsL*, which confer high-level streptomycin resistance, can cause a reduction in fitness of up to 26% (55). Similarly, expression of the 16S rRNA methyltransferase ArmA or mutations in the 16S rRNA, both increasing aminoglycoside resistance, are associated with a significant reduction in fitness (56, 57).

Compared to the fitness costs discussed above, expression of Arp1 in E. coli causes a similar reduction in fitness, and during exponential growth in nutrient-rich growth medium fitness is reduced by 36%. Under these conditions, clones expressing this peptide would be rapidly outcompeted, and fixation of the new function would be unlikely. However, in the presence of aminoglycoside antibiotics it confers a strong fitness advantage, allowing cell proliferation at concentrations which inhibit growth of the wild type. Thus, expression of these peptides offers, similar to SCVs, a conditional benefit to the bacterial cell. Interestingly, membrane depolarization via peptides has a distinct advantage over electron transport-defective mutants, in that expression and, therefore, the phenotype could evolve to become inducible, for example, by evolution of a regulated promoter or a regulatory small RNA. It is possible that endogenous, tightly regulated toxic peptides like TisB have evolved in this manner to induce growth arrest in environments where an active metabolism and cell division are detrimental (58, 59). Furthermore, similar to what has been observed for costly antibiotic resistance mutations, compensatory evolution could reduce the deleterious fitness effects and increase the chance of fixation.

Various studies have identified potential *de novo* genes in all domains of life based on comparative genomics (13, 60, 61), and experimental studies have shown that new functions can be selected by randomization of amino acid stretches located in preexisting structural scaffolds (62, 63). Recently, even variants with enzymatic functions could be isolated from a four-helix-bundle template (64). These studies demonstrate persuasively that new functions can arise from localized randomization, but they do not sufficiently address the functionality encoded in completely random sequence pools that do not rely on preexisting scaffold. Another study using unbiased sequence libraries to select ATP binding activity *in vitro* enriched four out of 6 × 10^12^ proteins with the selected activity (65). However, this experimental setup does not incorporate mRNA and protein stability *in vivo* (i.e., in a living bacterial cell) or that a beneficial function was conferred to the host. Similarly, a study utilizing biased expression libraries composed of glutamine, asparagine, and arginine residues *in vivo* showed that a large fraction is stably expressed, forms α-helices, and is protected from proteolytic degradation (66), but these studies did not reveal any beneficial selectable function for the bacterial cell. Here, we present experimental selection for peptides that confer a beneficial functionality *in vivo* and that were generated from randomized nucleotide sequences, supporting the idea that expression of randomly occurring sORFs can serve as a substrate for evolution of *de novo* genes. Further work is needed to determine what fraction of sequence space can generate such beneficial functions and which factors constrain how rapidly a proto-peptide/protein can be fine-tuned in its cellular function and how the initial fitness costs can be reduced. It would be interesting to examine if a cell can evolve to reduce the deleterious side effects of Arp expression. Evolution of such variants would likely require multiple genetic alterations which could be achieved with large bacterial populations during long-term evolution experiments in laboratory settings.

Finally, an interesting question is whether Arp-like proteins could provide aminoglycoside resistance in real-life clinical isolates. To our knowledge no one has searched for such functions, but we find it unlikely that they would be significant contributors, because there are other mechanisms that are easier to acquire and less deleterious to the cell. However, it is interesting to speculate that if Arps were present on plasmids and acquired regulatory elements, this could allow horizontal transfer and a conditionally inducible temporary reduction of membrane potential and associated resistance.

## MATERIALS AND METHODS

### Strains and growth conditions.

Most strains used in this study were derived from Escherichia coli K-12 BW25113 (designated E. coli throughout the text) and are listed in Table S1 in the supplemental material. If not indicated otherwise, Muller Hinton broth (MHB) (Becton, Dickinson) was used for growth in liquid cultures and Muller Hinton agar (MHA; MHB supplemented with 15 g/liter agar) for growth on plates. To maintain the plasmid pRD2 or pBAD18, the growth media were supplemented with 100 mg/liter ampicillin or 15 mg/liter chloramphenicol, respectively. Induction of expression was performed in the presence of 1 mM IPTG (pRD2) or 0.2% arabinose (pBAD18). For counterselection of *sacB*, no-salt lysogeny agar plates (10 g/liter tryptone, 5 g/liter yeast extract, 15 g/liter agar, and 50 g/liter sucrose) were used. Recovery after transformation was done in SOC medium (20 g/liter tryptone, 5 g/liter yeast extract, 0.5 g/liter NaCl, 10 mM MgCl_2_, 0.25 mM KCl, and 4 g/liter glucose). For washing and dilution of cells, phosphate-buffered saline (PBS; 8 g/liter NaCl, 0.2 g/liter KCl, 1.44 g/liter Na_2_HPO_4_, and 0.24 g/liter KH_2_PO_4_) was used.

### Library construction.

The randomized sequences were ordered as oligonucleotide pools from MWG Eurofins, IDT, and BioSyn. The oligonucleotide design for all libraries was similar, with a random sequence (composition is depicted in Fig. 1) flanked by fixed sequences upstream (TACGCTGGATCCATG) and downstream (TAACTAAGTAACTGCAGGCTTA). The oligonucleotides were complemented in a 1-cycle PCR using Phusion DNA polymerase and the complementation primer TAAGCCTGCAGTTACTTAG. The protocol consisted of 3 min of denaturation at 98°C followed by 5 min of annealing at 55°C and a 5-min elongation at 72°C. The plasmid pRD2 was isolated using the NucleoBond Xtra Midi kit (Macherey-Nagel) according to the manufacturer’s recommendations. To remove residual chromosomal DNA, the plasmid preparation was treated with Plasmid-Safe ATP-dependent DNase (Epicentre). The plasmid and double-stranded library were subsequently digested using the FastDigest restriction enzymes BamHI and PstI according to the manufacturer’s recommendations (ThermoFisher) and purified using a GeneJET gel extraction micro kit (ThermoFisher). The inserts were ligated into the vector using Ready-To-Go T4 DNA ligase (GE Healthcare) at a molar ratio of 4 inserts per vector. Purified ligations were mixed with electrocompetent NEB 5-alpha E. coli (New England BioLabs), transferred to an electroporation cuvette (1 mm gap), and electroporated using a Gene Pulser Xcell (Bio-Rad) at 1.8 kV, 400 Ω, and 25 μF. Cells were immediately recovered in 1 ml prewarmed SOC medium at 37°C with shaking at 200 rpm. After 1 h, a dilution series was plated on MHA containing 100 mg/liter ampicillin to determine the transformation efficiency (which equals the diversity of the cloned library). The remaining culture was split up into 300-μl aliquots, spread on MHA containing 100 mg/liter ampicillin, and incubated overnight at 37°C. The cells were scraped from the plates and plasmid libraries were extracted using a NucleoBond Xtra Midi kit (Macherey-Nagel).

To generate electrocompetent cells of E. coli BW25113, 1 ml culture was grown in MHB overnight and diluted 1:100 in prewarmed MHB. Cells were grown to an optical density at 600 nm (OD_600_) of 0.2 and rapidly cooled down in ice water for 10 min. The cells were pelleted by centrifugation at 4,000 × *g* and 4°C for 10 min and resuspended in ice-cold 10% glycerol. After 2 additional washing steps the pellet was resuspended in 10% glycerol, and 40 μl competent cells was premixed with approximately 200 ng of plasmid library in a chilled Eppendorf tube. The cell-DNA mixture was transferred to an electroporation cuvette and electroporated. Electroporation, recovery, and plating were performed as described above. The scraped cells were not subjected to plasmid extraction but instead mixed 1:1 with 50% glycerol and cryopreserved at −80°C in 100-μl aliquots, which contained approximately 2 × 10^10^ CFU per ml.

### Selection of antibiotic-resistant ORFs.

Six aliquots containing the different libraries and an empty vector control strain were thawed on ice, and 50 μl was transferred to 20 ml MHB containing 100 mg/liter ampicillin and 1 mM IPTG. Cultures were incubated for 3 h, and 200 μl was spread on MHA containing 100 mg/liter ampicillin, 1 mM IPTG, and the antibiotics used for screening of functional ORFs. Plates were incubated overnight at 37°C. Since selection was typically performed slightly above the MIC of an empty vector control strain, background colonies were frequently encountered due to inoculum effects or chromosomal mutants with elevated MICs. To distinguish between these background effects and increased resistance mediated by *de novo* ORFs, we scraped all colonies of the initial selection plates and extracted the plasmids using EZNA plasmid DNA minikit I (Omega) according to the manufacturer’s recommendations. The plasmids were then retransformed into E. coli BW25113 as described previously, recovered for 2 h in MHB containing 1 mM IPTG, and plated on MHA with 100 mg/liter ampicillin, 1 mM IPTG, and the screening antibiotic at the same concentration as that used for the initial selection. If the amount of colonies was similar to that in the initial selection, plates were discarded and this combination of antibiotic/concentration was no longer pursued. In cases were increased resistance was mediated by a plasmid-based ORF, the plasmid was enriched, resulting in a much higher abundance of resistant colonies for that particular selection. From these plates, we isolated individual clones and extracted the plasmid using EZNA plasmid DNA minikit I (Omega) according to the manufacturer’s recommendations. Plasmids were sequenced (primer, CCACCTGACGTCTAAGAA) and retransformed into E. coli BW25113. After recovery, the cells were spread on MHA with 100 mg/liter ampicillin. An individual clone was isolated and cryopreserved in 10% dimethyl sulfoxide (DMSO). This clone was subsequently tested for growth on plates containing the antibiotic from the initial selection. Additionally, we confirmed that growth was not observed if no IPTG was present in the plate to unambiguously show that the phenotype is caused by the randomly generated ORF in the plasmid under the inducible P*_LlacO_* promoter.

### Cloning using annealed oligonucleotides.

The insert of interest was designed *in silico*, and two complementary oligonucleotides with the desired overhangs after annealing were ordered from Eurofins or Sigma-Aldrich. Oligonucleotides were diluted to 1 pmol/μl in annealing buffer (10 mM Tris, pH 7.5, 100 mM NaCl, 100 μM EDTA) and mixed 1:1 in an Eppendorf tube to a final volume of 100 μl. The mixture was placed into boiling water and allowed to cool down to room temperature. The annealed oligonucleotides were directly used for cloning into the desired vector with an insert-to-plasmid ratio of 3:1. Ligations were performed using Ready-To-Go T4 DNA ligase (GE Healthcare) according to the manufacturer’s recommendations. After overnight incubation at 16°C, the reactions were cleaned and transformed as previously described.

### MIC determination.

MICs were determined using Etest strips (bioMérieux). Overnight cultures of the strains of interest were diluted 1:20 in PBS and spread on an MHA plate containing 100 mg/liter ampicillin and 1 mM IPTG where necessary with a cotton swab. Etest strips were applied in the middle of the plates and incubated at 37°C overnight. MIC values were scored after 20 h of incubation. Colistin MICs were determined using microdilution. For this, bacteria were grown on MHA with 100 mg/liter ampicillin, and 1 to 2 colonies were dissolved in 1 ml PBS to approximately 0.5 McFarland. The cell suspension was diluted 1:100 in Muller Hinton 2 broth (cation adjusted), and 50 μl was mixed with 50 μl Muller Hinton 2 broth containing 0.4% arabinose, 30 mg/liter chloramphenicol, and a 2-fold dilution series of colistin. The assay was performed in round-bottomed 96-well plates at 37°C overnight in a stationary position. The MIC was determined as the concentration where no growth could be observed and is based on a minimum of two biological replicates. If the determined values of the replicates were not identical, a third replicate was performed.

### Growth rate determination.

Exponential growth rates were determined with a Bioscreen C reader (Oy Growth Curves AB Ltd., Finland). For this, cultures were grown overnight in MH supplemented with 100 mg/liter ampicillin and diluted 1:1,000 in fresh medium. From the dilution, two 300-μl aliquots were transferred into honeycomb plates. The plates were incubated in a Bioscreen C reader for 16 h at 37°C with continuous shaking. Every 4 min the OD_600_ was measured, and the interval between 0.024 and 0.09 was used to determine the slope of the growth curve. All values are based on at least 4 biological replicates with two technical replicates each and are represented as relative to the growth of an empty vector control strain.

### Quantification of subpopulations with various resistance levels.

Overnight cultures were diluted in PBS to 3 × 10^3^ CFU/ml. One hundred μl of the cell suspension was plated on MHA plates containing 0, 0.75, 1.5, 3, and 6 mg/liter kanamycin. Additionally, all plates contained either 100 mg/liter ampicillin and 1 mM IPTG for strains carrying pRD2 variants or 15 mg/liter chloramphenicol and 0.2% arabinose for strains carrying pBAD18 variants. The plates were incubated overnight and colonies were counted. The frequencies of the subpopulations that were able to tolerate different kanamycin concentrations are relative to those of the colonies counted on plates without kanamycin.

### Sequence analysis.

Sequence design and analysis were performed using CLC Main Workbench (Qiagen). The peptides were aligned using Clustal Omega (67) with default parameters (Dealign input sequence, no; MBED-like clustering guide-tree, yes; MBED-like clustering iteration, yes; number of combined iterations, default [0]; maximum guide tree iterations, default; maximum hmm iterations, default; order, aligned). Transmembrane helices were predicted using the web tool TMHMM (68). BLAST searches were conducted using NCBI pBLAST with adjusted parameters for short input sequences (expect threshold, 200,000; word size, 2; matrix, PAM30; compositional adjustments, no adjustment). Grand average of hydropathy (GRAVY) values were determined with ExPASy ProtParam (69).

### Site-directed mutagenesis.

The site saturation mutagenesis was performed with GeneArt (ThermoFisher) based on the sequence of Arp1, where each position in the wild-type sequence was replaced by the amino acids lysine (K), glutamic acid (E), proline (P), tryptophan (W), and serine (S). All variants were subcloned into our modified expression vector, pBAD18-RND, using the AgeI/HindIII cloning sites, transformed into E. coli, and delivered as a total pool glycerol stock. The stock was plated out on MHA with 15 mg/liter chloramphenicol, and single colonies were restreaked on identical plates. From each clone, 2-ml overnight cultures were grown and plasmids were extracted using EZNA plasmid DNA minikit I (Omega). The plasmid variants were determined by Sanger sequencing (Eurofins, Germany). Nonredundant variants were transformed into E. coli BW25113 and subjected to MIC determination. A total of 300 clones were isolated from the pool in this manner, and variants that were not present in this set were constructed with a targeted approach using cloning of annealed oligonucleotides as described above.

### Immunogold TEM.

E. coli cells expressing Arp1-His tag or carrying an empty vector control were fixed for 45 min in 8% paraformaldehyde (PFA) plus 0.02% glutaraldehyde (GA) and MHB (1:1) in a 50-ml vial. The suspension was transferred to Eppendorf tubes (1.5 ml) and spun down, and the supernatant was replaced with 4% PFA plus 0.01% GA and fixed for a further 2 h. The cells were then dehydrated in alcohol and processed for embedding in K4M (Lowicryl), polymerized with UV light at −35°C in 72 h. All steps were performed at room temperature, except where otherwise stated. Ultrathin sections were cut in a Leica UCT Ultramicrotome and collected on gold grids. The immunostaining was done shortly after sectioning to improve access to epitope. Sections on grids were first blocked for 30 min in 2% bovine serum albumin in Tris-buffered saline, pH 7.4 (TBS; 6.05 g/liter Tris, 8.76 g/liter NaCl, pH adjusted with 1 M HCl); this was followed by incubation overnight at 20°C with the primary antibody (THE His tag antibody, monoclonal antibody, mouse; GenScript) diluted 1:5, 1:50, and 1:500 in TBS, pH 7.4. The grids then were rinsed three times in TBS, pH 7.4, followed by once in TBS, pH 8.3. Bound antibodies were localized by incubating the sections for 2 to 3 h on anti-mouse, 12-nm Au particles diluted 1:80. Finally, grids were rinsed three times in TBS, pH 7.4, and sterile H_2_O before being stained with 5% aqueous uranyl acetate and lead citrate. The grids were placed on drops of the respective reagent. Between all steps the grids were dried using filter paper. All incubations were carried out at room temperature. Grids were examined by a transmission electron microscope (TEM; FEI Tecnai G2) operated at 80 kV.

### Membrane potential assay.

Bacteria were grown overnight in MHB supplemented with 15 mg/liter chloramphenicol at 37°C. The following day all cultures were diluted 1:100 in MHB supplemented with 15 mg/liter chloramphenicol and grown to an OD_600_ of approximately 0.3 (time point 0). At the target OD_600_, IPTG was added to each culture to a final concentration of 1 mM, and cells were incubated for another 60 min to allow expression of proteins (time point 1). At both time points, approximately 10^6^ cells were transferred to 1 ml PBS with 10 mg/liter DiBAC_4_(3) (D8189; Sigma-Aldrich) with or without 100 μM carbonyl cyanide 3-chlorophenylhydrazone (CCCP; Sigma-Aldrich) and incubated for 30 min at room temperature in the dark. Fluorescence was measured with a MACSQuant VYB (Miltenyi Biotec) using the GFP/FITC (green fluorescent protein/fluorescein isothiocyanate) channel and a threshold of 15,000 gated cells. The raw data were analyzed and visualized with FlowJo (BD Bioscience).

### Radiolabeled dihydrostreptomycin uptake assay.

Overnight cultures were diluted 1:200 in MHB containing 15 mg/liter chloramphenicol and grown to an OD_600_ of 0.6 at 37°C with continuous shaking. After reaching the target OD_600_, a 10% arabinose solution was added to a final concentration of 0.2% and incubated for 10 min to allow expression. The cultures were then supplemented with tritiated dihydrostreptomycin (Larodan, Sweden) to a final activity of 200 nCi/ml. Control cultures were additionally supplemented with CCCP to a final concentration of 100 μM. After 20 min, 2-ml aliquots of the cultures were transferred to Eppendorf tubes and pelleted by centrifugation at 13,500 × *g* for 3 min, and the supernatant was decanted. The pellets were washed twice with PBS. After the last wash, the pellets were dissolved in 1 ml PBS and transferred to 2 ml Optiphase HiSafe 3 scintillation cocktail (PerkinElmer). The samples were measured with a Tri-Carb 2810 TR liquid scintillation analyzer over a duration of 1 min per sample. The counts were normalized to OD_600_ values determined after the 20-min incubation with dihydrostreptomycin to correct for differences in cell density. The counts of the CCCP control were subtracted from those of the other samples to correct for uptake in cells with a completely collapsed membrane potential and binding of dihydrostreptomycin to the cell surface.

### Selection of chromosomal mutants.

Four independent cultures of E. coli BW25113 carrying the empty pRD2 control vector were grown overnight in MHB with 100 mg/liter ampicillin and plated on MHA with 100 mg/liter ampicillin, 1 mM IPTG, and either 6 mg/liter kanamycin or amikacin. Plates were incubated overnight, and resistant mutants were purified on plates containing the same amount of antibiotic. Overnight cultures were grown in MHB with 100 mg/liter ampicillin and cryopreserved in 10% DMSO. To test the additivity of the chromosomal mutants with overexpression of Arp1, the empty control plasmid was cured. For this, cultures were grown overnight in MHB without ampicillin and plated on no-salt LA plates containing 5% sucrose for counterselection of *sacB*. Colonies were restreaked on identical plates, and plasmid loss was confirmed by testing for ampicillin susceptibility. Plasmid-free strains were then subjected to transformation as described previously.

### Whole-genome sequencing.

Bacteria were grown overnight, and 500-μl samples were used for the preparation of genomic DNA using the MasterPure complete DNA and RNA purification kit (Epicentre) according to the manufacturer’s recommendations. In brief, cells were pelleted by centrifugation for 2 min at 13,500 × *g*. The supernatant was removed and the pellet resuspended in 300 μl tissue and cell lysis solution (Epicentre) containing 50 μg proteinase K. After 15 min of incubation at 65°C, the sample was cooled to 37°C and 5 μg RNaseA was added, followed by another incubation for 30 min at 37°C. A volume of 175 μl MPC protein precipitation reagent (Epicentre) was added to the lysed sample and vortexed vigorously. Debris was pelleted by centrifugation for 20 min at 13,500 × *g* and the supernatant transferred to an Eppendorf tube. The genomic DNA was precipitated by addition of 500 μl isopropanol and washed twice with 200 μl 75% ethanol by repeated centrifugation. Finally, the DNA was resuspended in water and diluted to a final concentration of 0.2 μg/liter. Illumina’s Nextera XT kit was used to generate libraries (2× 300) to be sequenced with MiSeq per the manufacturer’s recommendations. All samples were dual-indexed and pooled. Average whole-genome coverage per sample was approximately 40-fold. Analysis of the fastq files obtained from MiSeq sequencing was performed using CLC Genomics Workbench. Single-nucleotide polymorphism (SNP) calling and structural variants were assessed using standard parameters. Raw sequence reads were deposited under BioProject identifier PRJNA460371.

### Purification of cytochrome *c* oxidase and proteoliposome preparation.

Cytochrome *c* oxidase (Cyt*c*O) was purified from bovine heart mitochondria as described before (70). Enzyme concentration was determined from a reduced-oxidized difference spectrum (400 to 700 nm) using the 603-nm peak and ε = 24 mM^−1^. Proteoliposomes were prepared essentially as described in reference 71. Briefly, l-α-phosphatidylcholine (PC) from soy beans (Sigma-Aldrich) was homogenized at 20 mg/ml in 20 mM HEPES, pH 7.5, 100 mM KCl, 50 g/liter sucrose. Large liposomes were formed by rapidly freezing the lipid solution for 1 min in liquid nitrogen, followed by thawing on a 30°C heat block with 30 s of vortexing between treatments. This procedure was repeated 5 times, and the liposomes were stored at −80°C. Small unilamellar vesicles (SUVs) were formed by extruding the liposomes with a miniextruder through 100-nm Whatman extruder membranes. Proteoliposomes were formed by adding 0.6% sodium cholate and 1.3 μl Cyt*c*O (at 215 μM) and dialyzing the sample overnight against 1,000 volumes of liposome buffer to remove the detergent. This yields, on average, 2 to 3 Cyt*c*Os per liposome.

### Peptide preparation.

The synthetic peptides were purchased from Biosynthesis. The dry peptides were suspended at 5 mg/ml in DMSO and sonicated for 5 min in a water sonication bath to make sure they were completely dissolved.

### Oxygen consumption assay.

Cyt*c*O activity was measured using a Clark-type oxygen electrode (Hansatech) at 25°C. In the measuring chamber, 1 ml 20 mM HEPES, pH 7.5, 100 mM KCl, 0.1 mM EDTA was mixed with 12 mM ascorbate, 70 μM TMPD, and 60 μM cytochrome *c* under constant stirring. Oxygen consumption rates and RCRs were measured essentially as described in reference 43. Briefly, the coupled rate for Cyt*c*O activity was calculated from the slope after addition of 20 μl proteoliposomes by subtracting the baseline slope. Subsequently, 30 μl peptide solution was added and the rate was again calculated (by subtracting the baseline). As positive controls, the electric component of the proton electrochemical gradient was dissipated by addition of 6 μM valinomycin, and the gradient was subsequently fully dissipated by addition of 21 μM FCCP. To test for direct putative effects by the peptides on the Cyt*c*O activity, oxygen consumption rates were also measured for protein solubilized in 0.05% dodecyl-β-maltoside (DDM) detergent.

### Data availability.

All data necessary to understand and evaluate the conclusions of this study are available in the paper and supplemental materials. Raw sequence reads of the mutants that were whole-genome sequenced are available at the BioProject database under BioProject number PRJNA460371. An annotated sequence file of the expression vector (pRD2) is available at GenBank under the accession number MH298521.
