# Supplementary figures and images for: De Novo Emergence of Peptides That Confer Antibiotic Resistance
Source: mBio. 2019 Jun 4;10(3):e00837-19. doi: 10.1128/mBio.00837-19 (PMC6550523; doi:10.1128/mBio.00837-19)

A

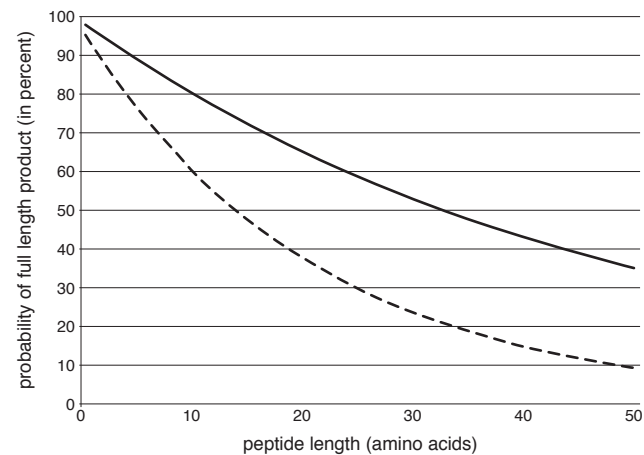

B

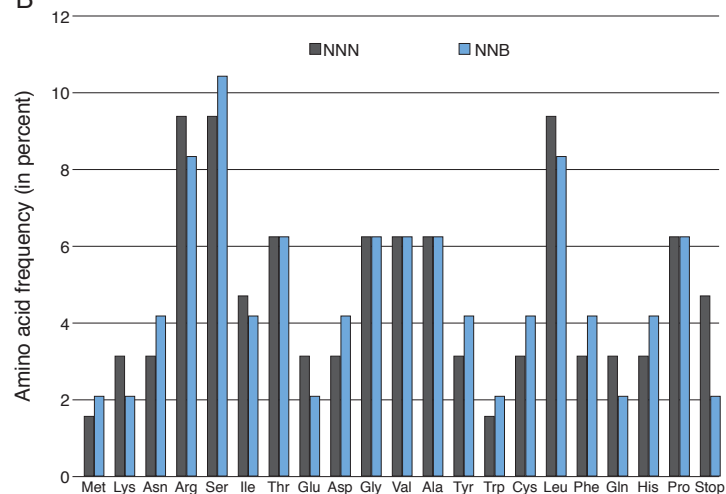

C

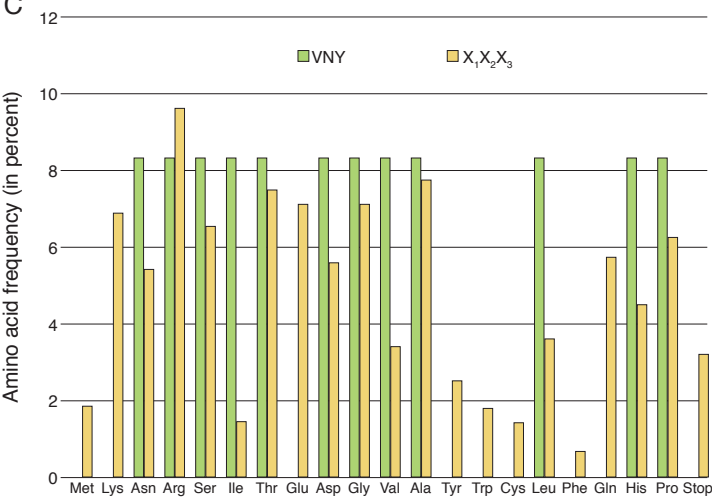

Supplement: FIG S1 [file mBio.00837-19-sf001.pdf]

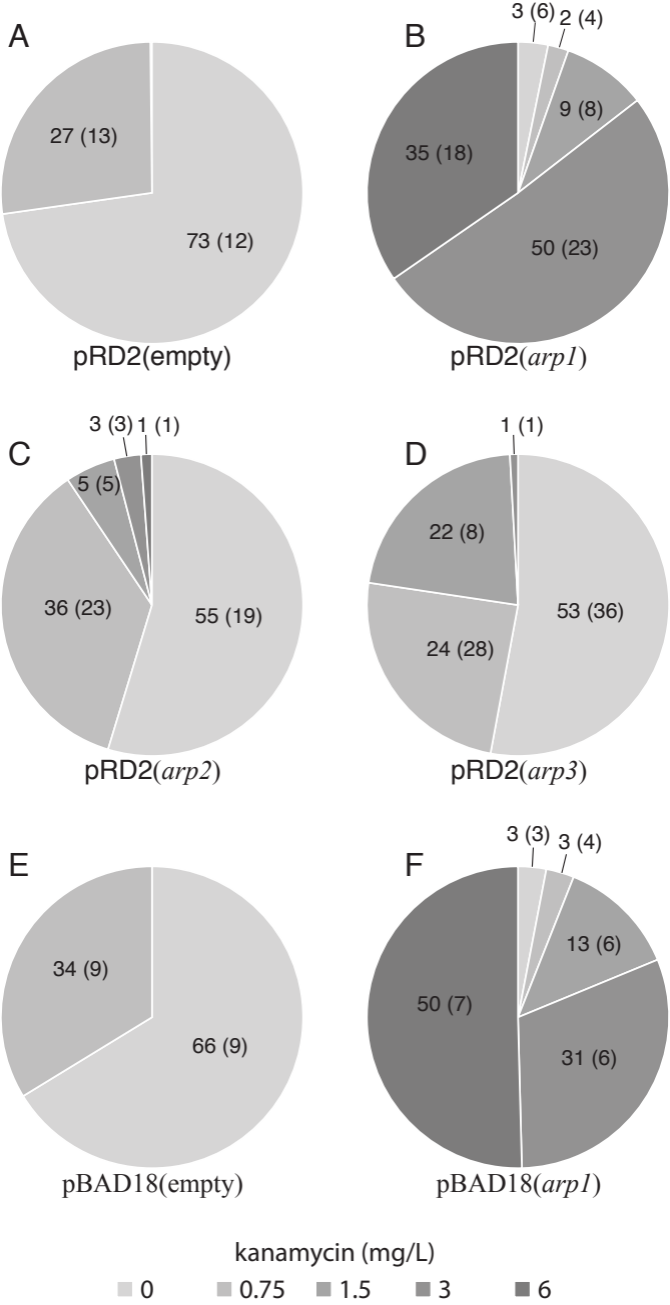

Supplement: FIG S2 [file mBio.00837-19-sf002.pdf]

A

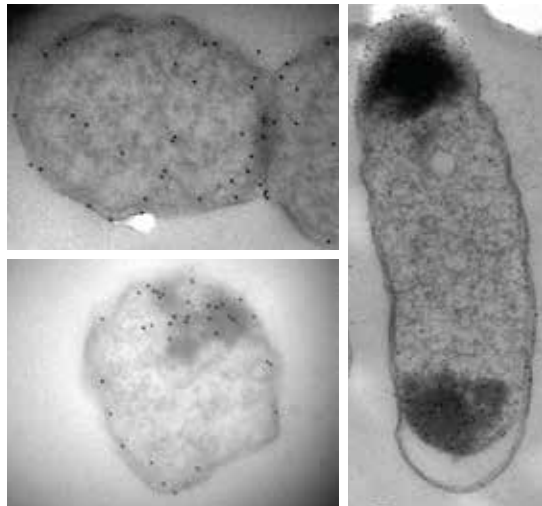

B

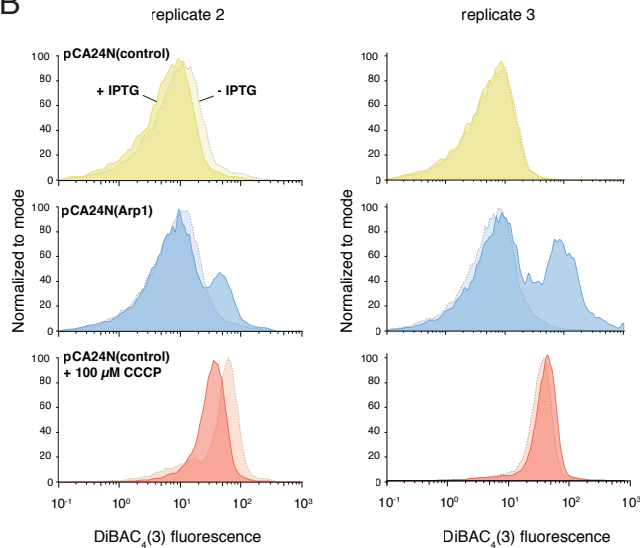

Supplement: FIG S5 [file mBio.00837-19-sf005.pdf]

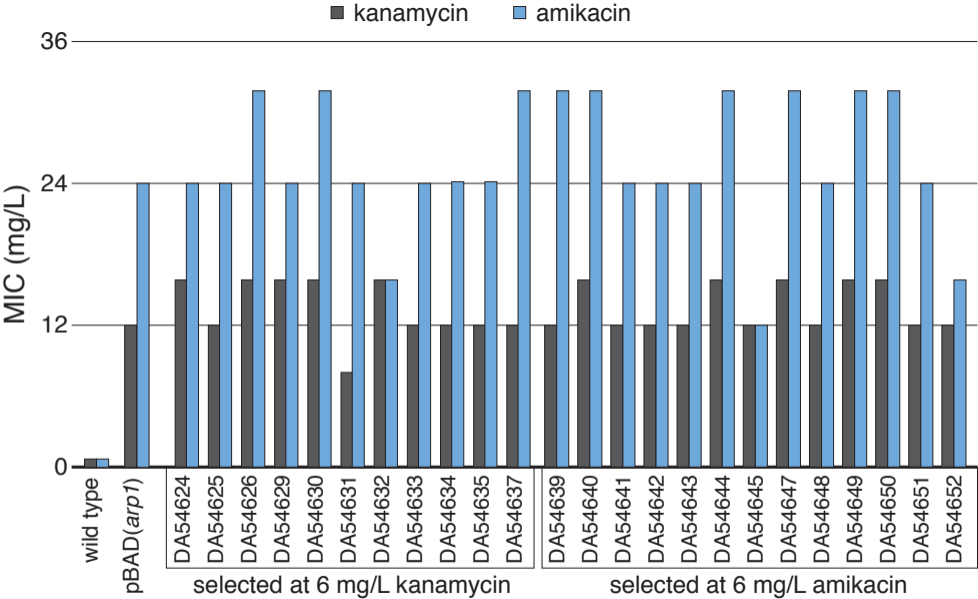

Supplement: FIG S3 [file mBio.00837-19-sf003.pdf]

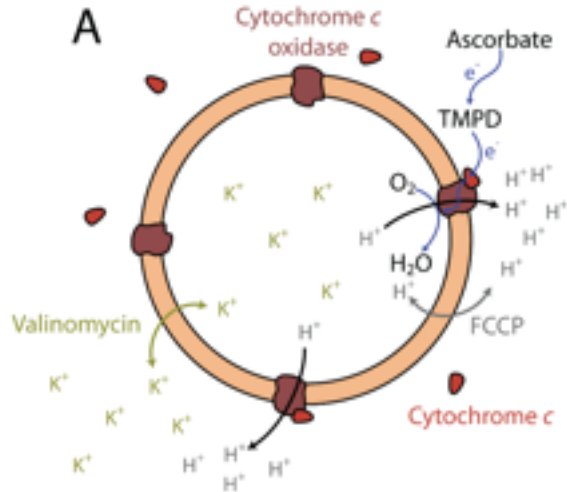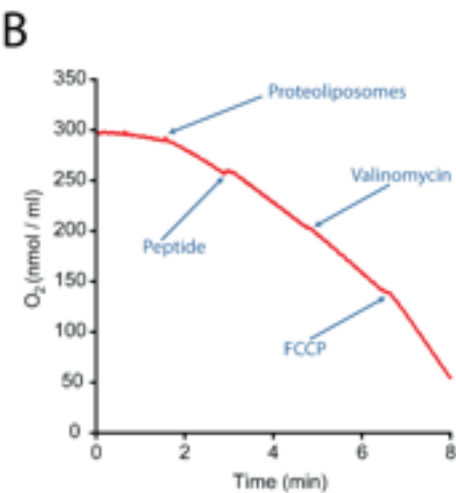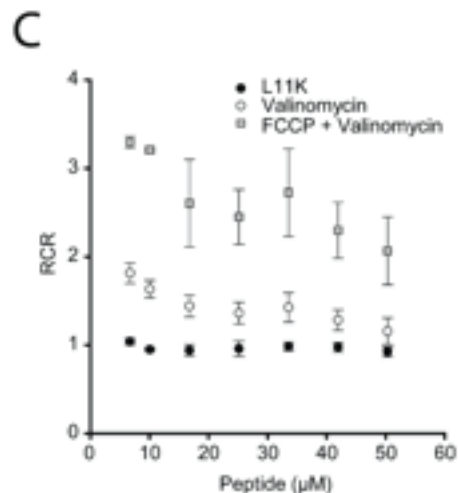

Supplement: FIG S4 [file mBio.00837-19-sf004.pdf]
