# Supplementary material for: De Novo Emergence of Peptides That Confer Antibiotic Resistance
Source: mBio. 2019 Jun 4;10(3):e00837-19. doi: 10.1128/mBio.00837-19 (PMC6550523; doi:10.1128/mBio.00837-19)
Supplement: TABLE S1 [file mBio.00837-19-st001.pdf]

Supplementary Table 1.

List of strains used in this study.

Strain number	Strain	Plasmid	Comment
DA28696	*E: coli* K-12 BW25113	-	
DA53311	*E: coli* K-12 BW25113	pRD2	
DA53969	*E: coli* K-12 BW25113	pRD2(*arp1*)	
DA53977	*E: coli* K-12 BW25113	pRD2(*arp2*)	
DA53979	*E: coli* K-12 BW25113	pRD2(*arp3*)	
DA57920	*E: coli* K-12 BW25113	pBAD18	
DA57921	*E: coli* K-12 BW25113	pBAD18(*arp1*)	
DA57369	*E: coli* K-12 BW25113	pBAD18(*arp1*-His)	
DA60068	*E: coli* K-12 BW25113	pCA24N	
DA60069	*E: coli* K-12 BW25113	pCA24N(*arp1*)	
DA5438	*E. coli* K-12 MG1655	-	
DA52893	*E. coli* K-12 MG1655	pBAD18	
DA55590	*E. coli* K-12 MG1655	pBAD18(*arp1*)	
DA62567	*E. coli* K-12 MG1655 *bglGFP*::PLlacO_*arp1*	-	
DA6192	*S. typhimurium* LT2	-	
DA55755	*S. typhimurium* LT2	pBAD18	
DA55756	*S. typhimurium* LT2	pBAD18(*arp1*)	
DA12755	*K. pneumoniae* ATCC13883	-	
DA57896	*K. pneumoniae* ATCC13883	pBAD18	
DA57955	*K. pneumoniae* ATCC13883	pBAD18(*arp1*)	
DA54624	*E. coli* K-12 BW25113	pRD2(empty)	selected on 6 mg/L kanamycin
DA54625	*E. coli* K-12 BW25113	pRD2(empty)	selected on 6 mg/L kanamycin
DA54626	*E. coli* K-12 BW25113	pRD2(empty)	selected on 6 mg/L kanamycin
DA54629	*E. coli* K-12 BW25113	pRD2(empty)	selected on 6 mg/L

			kanamycin
DA54630	*E. coli* K-12 BW25113	pRD2(empty)	selected on 6 mg/L kanamycin
DA54631	*E. coli* K-12 BW25113	pRD2(empty)	selected on 6 mg/L kanamycin
DA54632	*E. coli* K-12 BW25113	pRD2(empty)	selected on 6 mg/L kanamycin
DA54633	*E. coli* K-12 BW25113	pRD2(empty)	selected on 6 mg/L kanamycin
DA54634	*E. coli* K-12 BW25113	pRD2(empty)	selected on 6 mg/L kanamycin
DA54635	*E. coli* K-12 BW25113	pRD2(empty)	selected on 6 mg/L kanamycin
DA54637	*E. coli* K-12 BW25113	pRD2(empty)	selected on 6 mg/L kanamycin
DA54639	*E. coli* K-12 BW25113	pRD2(empty)	selected on 6 mg/L amikacin
DA54640	*E. coli* K-12 BW25113	pRD2(empty)	selected on 6 mg/L amikacin
DA54641	*E. coli* K-12 BW25113	pRD2(empty)	selected on 6 mg/L amikacin
DA54642	*E. coli* K-12 BW25113	pRD2(empty)	selected on 6 mg/L amikacin
DA54643	*E. coli* K-12 BW25113	pRD2(empty)	selected on 6 mg/L amikacin
DA54644	*E. coli* K-12 BW25113	pRD2(empty)	selected on 6 mg/L amikacin
DA54645	*E. coli* K-12 BW25113	pRD2(empty)	selected on 6 mg/L amikacin
DA54647	*E. coli* K-12 BW25113	pRD2(empty)	selected on 6 mg/L amikacin
DA54648	*E. coli* K-12 BW25113	pRD2(empty)	selected on 6 mg/L amikacin
DA54649	*E. coli* K-12 BW25113	pRD2(empty)	selected on 6 mg/L amikacin
DA54650	*E. coli* K-12 BW25113	pRD2(empty)	selected on 6 mg/L amikacin
DA54651	*E. coli* K-12 BW25113	pRD2(empty)	selected on 6 mg/L amikacin
DA54652	*E. coli* K-12 BW25113	pRD2(empty)	selected on 6 mg/L amikacin
DA54853	*E. coli* K-12 BW25113	-	DA54632 plasmid cured
DA57926	*E. coli* K-12 BW25113	pBAD18	
DA57927	*E. coli* K-12 BW25113	pBAD18(*arp1*)	
DA54847	*E. coli* K-12 BW25113	-	DA54626 plasmid cured
DA57922	*E. coli* K-12 BW25113	pBAD18	

DA57923	*E. coli* K-12 BW25113	pBAD18(*arp1*)	
DA54850	*E. coli* K-12 BW25113	-	DA54629 plasmid cured
DA57924	*E. coli* K-12 BW25113	pBAD18	
DA57925	*E. coli* K-12 BW25113	pBAD18(*arp1*)	
DA54862	*E. coli* K-12 BW25113	-	DA54641 plasmid cured
DA58136	*E. coli* K-12 BW25113	pBAD18	
DA58137	*E. coli* K-12 BW25113	pBAD18(*arp1*)	
DA54869	*E. coli* K-12 BW25113	-	DA54648 plasmid cured
DA58140	*E. coli* K-12 BW25113	pBAD18	
DA58141	*E. coli* K-12 BW25113	pBAD18(*arp1*)	
DA54871	*E. coli* K-12 BW25113	-	DA54650 plasmid cured
DA58144	*E. coli* K-12 BW25113	pBAD18	
DA58145	*E. coli* K-12 BW25113	pBAD18(*arp1*)	
DA49828	*E. coli* K-12 MG1655 *rpsL* K42N	-	
DA56513	*E. coli* K-12 MG1655 *rpsL* K42N	pBAD18	
DA56514	*E. coli* K-12 MG1655 *rpsL* K42N	pBAD18(*arp1*)	
DA40134	*S. typhimurium* LT2 *gidB* Q167*	-	
DA57865	*S. typhimurium* LT2 *gidB* Q167*	pBAD18	
DA57866	*S. typhimurium* LT2 *gidB* Q167*	pBAD18(*arp1*)	
DA62112	*E. coli* K-12 BW25113	pBAD18(*envZ-TM1)*	
DA62113	*E. coli* K-12 BW25113	pBAD18(*envZ-TM2)*	
DA62114	*E. coli* K-12 BW25113	pBAD18(*phoQ-TM1)*	
DA62115	*E. coli* K-12 BW25113	pBAD18(*phoQ-TM2)*	
DA62116	*E. coli* K-12 BW25113	pBAD18(*pstC-TM1)*	
DA62117	*E. coli* K-12 BW25113	pBAD18(*pstC-TM2)*	
DA62118	*E. coli* K-12 BW25113	pBAD18(*murP-TM1)*	
DA62119	*E. coli* K-12 BW25113	pBAD18(*murP-TM2)*	
DA62120	*E. coli* K-12 BW25113	pBAD18(*pitA-TM1)*	
DA62121	*E. coli* K-12 BW25113	pBAD18(*pitA-TM2)*	
DA58975	*E: coli* K-12 BW25113	pBAD18(*arp1*)	Mutagenesis variant L2K
DA58072	*E: coli* K-12 BW25113	pBAD18(*arp1*)	Mutagenesis variant L2E
DA58085	*E: coli* K-12 BW25113	pBAD18(*arp1*)	Mutagenesis variant L2P
DA58104	*E: coli* K-12 BW25113	pBAD18(*arp1*)	Mutagenesis variant L2W

DA58098	*E: coli* K-12 BW25113	pBAD18(*arp1*)	Mutagenesis variant L2S
DA58638	*E: coli* K-12 BW25113	pBAD18(*arp1*)	Mutagenesis variant L3K
DA58733	*E: coli* K-12 BW25113	pBAD18(*arp1*)	Mutagenesis variant L3E
DA58650	*E: coli* K-12 BW25113	pBAD18(*arp1*)	Mutagenesis variant L3P
DA58728	*E: coli* K-12 BW25113	pBAD18(*arp1*)	Mutagenesis variant L3W
DA58646	*E: coli* K-12 BW25113	pBAD18(*arp1*)	Mutagenesis variant L3S
DA58976	*E: coli* K-12 BW25113	pBAD18(*arp1*)	Mutagenesis variant F4K
DA58097	*E: coli* K-12 BW25113	pBAD18(*arp1*)	Mutagenesis variant F4E
DA58101	*E: coli* K-12 BW25113	pBAD18(*arp1*)	Mutagenesis variant F4P
DA58090	*E: coli* K-12 BW25113	pBAD18(*arp1*)	Mutagenesis variant F4W
DA58657	*E: coli* K-12 BW25113	pBAD18(*arp1*)	Mutagenesis variant F4S
DA58635	*E: coli* K-12 BW25113	pBAD18(*arp1*)	Mutagenesis variant F5K
DA58648	*E: coli* K-12 BW25113	pBAD18(*arp1*)	Mutagenesis variant F5E
DA58652	*E: coli* K-12 BW25113	pBAD18(*arp1*)	Mutagenesis variant F5P
DA58639	*E: coli* K-12 BW25113	pBAD18(*arp1*)	Mutagenesis variant F5W
DA58119	*E: coli* K-12 BW25113	pBAD18(*arp1*)	Mutagenesis variant F5S
DA58106	*E: coli* K-12 BW25113	pBAD18(*arp1*)	Mutagenesis variant C6K
DA58653	*E: coli* K-12 BW25113	pBAD18(*arp1*)	Mutagenesis variant C6E
DA58980	*E: coli* K-12 BW25113	pBAD18(*arp1*)	Mutagenesis variant C6P
DA58068	*E: coli* K-12 BW25113	pBAD18(*arp1*)	Mutagenesis variant C6W
DA58084	*E: coli* K-12 BW25113	pBAD18(*arp1*)	Mutagenesis variant C6S
DA58074	*E: coli* K-12 BW25113	pBAD18(*arp1*)	Mutagenesis variant F7K
DA58734	*E: coli* K-12 BW25113	pBAD18(*arp1*)	Mutagenesis variant F7E
DA58727	*E: coli* K-12 BW25113	pBAD18(*arp1*)	Mutagenesis variant F7P
DA58086	*E: coli* K-12 BW25113	pBAD18(*arp1*)	Mutagenesis variant F7W
DA58109	*E: coli* K-12 BW25113	pBAD18(*arp1*)	Mutagenesis variant F7S
DA58103	*E: coli* K-12 BW25113	pBAD18(*arp1*)	Mutagenesis variant I8K
DA58077	*E: coli* K-12 BW25113	pBAD18(*arp1*)	Mutagenesis variant I8E
DA58737	*E: coli* K-12 BW25113	pBAD18(*arp1*)	Mutagenesis variant I8P
DA58091	*E: coli* K-12 BW25113	pBAD18(*arp1*)	Mutagenesis variant I8W
DA58107	*E: coli* K-12 BW25113	pBAD18(*arp1*)	Mutagenesis variant I8S
DA58116	*E: coli* K-12 BW25113	pBAD18(*arp1*)	Mutagenesis variant F9K
DA58113	*E: coli* K-12 BW25113	pBAD18(*arp1*)	Mutagenesis variant F9E

DA58118	*E: coli* K-12 BW25113	pBAD18(*arp1*)	Mutagenesis variant F9P
DA58087	*E: coli* K-12 BW25113	pBAD18(*arp1*)	Mutagenesis variant F9W
DA58111	*E: coli* K-12 BW25113	pBAD18(*arp1*)	Mutagenesis variant F9S
DA58736	*E: coli* K-12 BW25113	pBAD18(*arp1*)	Mutagenesis variant L10K
DA58979	*E: coli* K-12 BW25113	pBAD18(*arp1*)	Mutagenesis variant L10E
DA58644	*E: coli* K-12 BW25113	pBAD18(*arp1*)	Mutagenesis variant L10P
DA58067	*E: coli* K-12 BW25113	pBAD18(*arp1*)	Mutagenesis variant L10W
DA58108	*E: coli* K-12 BW25113	pBAD18(*arp1*)	Mutagenesis variant L10S
DA58731	*E: coli* K-12 BW25113	pBAD18(*arp1*)	Mutagenesis variant L11K
DA58105	*E: coli* K-12 BW25113	pBAD18(*arp1*)	Mutagenesis variant L11E
DA58117	*E: coli* K-12 BW25113	pBAD18(*arp1*)	Mutagenesis variant L11P
DA58641	*E: coli* K-12 BW25113	pBAD18(*arp1*)	Mutagenesis variant L11W
DA58982	*E: coli* K-12 BW25113	pBAD18(*arp1*)	Mutagenesis variant L11S
DA58735	*E: coli* K-12 BW25113	pBAD18(*arp1*)	Mutagenesis variant I12K
DA58651	*E: coli* K-12 BW25113	pBAD18(*arp1*)	Mutagenesis variant I12E
DA58081	*E: coli* K-12 BW25113	pBAD18(*arp1*)	Mutagenesis variant I12P
DA58080	*E: coli* K-12 BW25113	pBAD18(*arp1*)	Mutagenesis variant I12W
DA58983	*E: coli* K-12 BW25113	pBAD18(*arp1*)	Mutagenesis variant I12S
DA58093	*E: coli* K-12 BW25113	pBAD18(*arp1*)	Mutagenesis variant V13K
DA58096	*E: coli* K-12 BW25113	pBAD18(*arp1*)	Mutagenesis variant V13E
DA58102	*E: coli* K-12 BW25113	pBAD18(*arp1*)	Mutagenesis variant V13P
DA58649	*E: coli* K-12 BW25113	pBAD18(*arp1*)	Mutagenesis variant V13W
DA58076	*E: coli* K-12 BW25113	pBAD18(*arp1*)	Mutagenesis variant V13S
DA58064	*E: coli* K-12 BW25113	pBAD18(*arp1*)	Mutagenesis variant W14K
DA58095	*E: coli* K-12 BW25113	pBAD18(*arp1*)	Mutagenesis variant W14E
DA58066	*E: coli* K-12 BW25113	pBAD18(*arp1*)	Mutagenesis variant W14P
DA58729	*E: coli* K-12 BW25113	pBAD18(*arp1*)	Mutagenesis variant W14S
DA58977	*E: coli* K-12 BW25113	pBAD18(*arp1*)	Mutagenesis variant L15K
DA58647	*E: coli* K-12 BW25113	pBAD18(*arp1*)	Mutagenesis variant L15E
DA58981	*E: coli* K-12 BW25113	pBAD18(*arp1*)	Mutagenesis variant L15P
DA58065	*E: coli* K-12 BW25113	pBAD18(*arp1*)	Mutagenesis variant L15W
DA58071	*E: coli* K-12 BW25113	pBAD18(*arp1*)	Mutagenesis variant L15S
DA58094	*E: coli* K-12 BW25113	pBAD18(*arp1*)	Mutagenesis variant C16K

DA58073	*E: coli* K-12 BW25113	pBAD18(*arp1*)	Mutagenesis variant C16E
DA58732	*E: coli* K-12 BW25113	pBAD18(*arp1*)	Mutagenesis variant C16P
DA58088	*E: coli* K-12 BW25113	pBAD18(*arp1*)	Mutagenesis variant C16W
DA58645	*E: coli* K-12 BW25113	pBAD18(*arp1*)	Mutagenesis variant C16S
DA58082	*E: coli* K-12 BW25113	pBAD18(*arp1*)	Mutagenesis variant I17K
DA58643	*E: coli* K-12 BW25113	pBAD18(*arp1*)	Mutagenesis variant I17E
DA58656	*E: coli* K-12 BW25113	pBAD18(*arp1*)	Mutagenesis variant I17P
DA58112	*E: coli* K-12 BW25113	pBAD18(*arp1*)	Mutagenesis variant I17W
DA58092	*E: coli* K-12 BW25113	pBAD18(*arp1*)	Mutagenesis variant I17S
DA58636	*E: coli* K-12 BW25113	pBAD18(*arp1*)	Mutagenesis variant L18K
DA58078	*E: coli* K-12 BW25113	pBAD18(*arp1*)	Mutagenesis variant L18E
DA58640	*E: coli* K-12 BW25113	pBAD18(*arp1*)	Mutagenesis variant L18P
DA58070	*E: coli* K-12 BW25113	pBAD18(*arp1*)	Mutagenesis variant L18W
DA58984	*E: coli* K-12 BW25113	pBAD18(*arp1*)	Mutagenesis variant L18S
DA58655	*E: coli* K-12 BW25113	pBAD18(*arp1*)	Mutagenesis variant A19K
DA58079	*E: coli* K-12 BW25113	pBAD18(*arp1*)	Mutagenesis variant A19E
DA58642	*E: coli* K-12 BW25113	pBAD18(*arp1*)	Mutagenesis variant A19P
DA58099	*E: coli* K-12 BW25113	pBAD18(*arp1*)	Mutagenesis variant A19W
DA58100	*E: coli* K-12 BW25113	pBAD18(*arp1*)	Mutagenesis variant A19S
DA58654	*E: coli* K-12 BW25113	pBAD18(*arp1*)	Mutagenesis variant F20K
DA58114	*E: coli* K-12 BW25113	pBAD18(*arp1*)	Mutagenesis variant F20E
DA58089	*E: coli* K-12 BW25113	pBAD18(*arp1*)	Mutagenesis variant F20P
DA58069	*E: coli* K-12 BW25113	pBAD18(*arp1*)	Mutagenesis variant F20W
DA58730	*E: coli* K-12 BW25113	pBAD18(*arp1*)	Mutagenesis variant F20S
DA58726	*E: coli* K-12 BW25113	pBAD18(*arp1*)	Mutagenesis variant R21K
DA58075	*E: coli* K-12 BW25113	pBAD18(*arp1*)	Mutagenesis variant R21E
DA58083	*E: coli* K-12 BW25113	pBAD18(*arp1*)	Mutagenesis variant R21P
DA58634	*E: coli* K-12 BW25113	pBAD18(*arp1*)	Mutagenesis variant R21W
DA58985	*E: coli* K-12 BW25113	pBAD18(*arp1*)	Mutagenesis variant R21S
DA58978	*E: coli* K-12 BW25113	pBAD18(*arp1*)	Mutagenesis variant S22K
DA58110	*E: coli* K-12 BW25113	pBAD18(*arp1*)	Mutagenesis variant S22E
DA58637	*E: coli* K-12 BW25113	pBAD18(*arp1*)	Mutagenesis variant S22P
DA58115	*E: coli* K-12 BW25113	pBAD18(*arp1*)	Mutagenesis variant S22W
