# Supplementary material for: De Novo Emergence of Peptides That Confer Antibiotic Resistance
Source: mBio. 2019 Jun 4;10(3):e00837-19. doi: 10.1128/mBio.00837-19 (PMC6550523; doi:10.1128/mBio.00837-19)
Supplement: TABLE S2 [file mBio.00837-19-st002.pdf]

Supplementary Table 2.

Minimal inhibitory concentrations of different antibiotics for strains carrying an empty vector control or expressing Arp1-3. Values represent the concentration in mg/L of the respective antibiotic that inhibited growth.

**Antibiotic**	**DA53311 /pRD2(empty)**	**DA53969 /pRD2(*arp*1)**	**DA53977 /pRD2(*arp*2)**	**DA53979 /pRD2(*arp*3)**
kanamycin	0.75	12	4	3
streptomycin	1	16	12	8
gentamycin	0.094	4	1.5	1
amikacin	0.5	24	8	6
colistin	0.5	0.25	0.5	0.5
chloramphenicol	8	4	6	6
ertapenem	0.004	0.006	0.004	0.004
erythromycin	32	48	48	48
cefaclor	12	24	12	12
ceftazidime	0.19	0.19	0.25	0.19
tetracycline	1.5	1	1	1
ciprofloxacin	0.008	0.012	0.008	0.008
trimethoprim	0.38	0.38	0.38	0.38
