# Supplementary material for: De Novo Emergence of Peptides That Confer Antibiotic Resistance
Source: mBio. 2019 Jun 4;10(3):e00837-19. doi: 10.1128/mBio.00837-19 (PMC6550523; doi:10.1128/mBio.00837-19)
Supplement: TABLE S3 [file mBio.00837-19-st003.pdf]

Supplementary Table 3.

Verification of hits. To ensure the phenotype was mediated by the encoded peptide, the level of resistance conferred after re-coding, induction by IPTG, removal of the start codon and introduction of a frame shift was determined. Growth on plates containing 6 mg/L kanamycin is indicated with '+', weak growth with '(+)' and no growth with '-'.

	**recloned**		**recoded**	**no ATG**	**frame shift**
	1mM IPTG	no IPTG			
arp1	+	-	(+)	-	-
arp2	+	-	ND	ND	ND
arp3	+	-	(+)	-	-

Supplementary Table 4.

Whole genome sequencing results of six chromosomal mutants selected on 6 mg/L kanamycin (DA54853, DA54847, DA54850) or amikacin (DA54862, DA54869, DA54871). Insertions of IS5 (*insH)*, IS1 (*insB)* and adenine (A) occurred upstream (us) of the corresponding gene.

DA-number	gene	substitution	insertion/deletion
DA54853	*cydA*		152us:IS5
DA54847	*abrB*	T293A	
	cydA	G266T	
	yffS		36us:A
DA54850	*potD*	A445T	
	mdtB	G1088A	
	ubiF		31_45del
DA54862	*ubiJ*		G270del
DA54869	*ubiF*		31_45del
DA54871	*cydA*		175us:IS1

Supplementary Table 5. List of transmembrane helices tested for increased aminoglycoside resistance.

Gene	Helix #	Sequence	Length (aa)	Increasing resistance
envZ	1	MLLLIVTLLFASLVTTYLVVL	21	No
	2	MPLFRYTLAIMLLAIGGAWLFI	22	No
phoQ	1	MFLLATAAVVLVLSLAYGMVAL	22	No
	2	MFIYVLSANLLLVIPLLWVAAW	22	No
pstC	1	MLAALIVLLMLGGIIVSLIIS	21	No
	2	MIYGTLVTSFIALLIAVPVSF	21	No
murP	1	MLIPGFIAAGLLLGIATLIATV	22	No
	2	MALNFMKVFSKGLFTFLVILVG	22	No
pitA	1	MFAGLDLHTGLLLLLALAFVLF	22	No
	2	MLAVVMAAVFNFLGVLLGGLSV	22	No
