# Supplementary material for: De Novo Emergence of Peptides That Confer Antibiotic Resistance
Source: mBio. 2019 Jun 4;10(3):e00837-19. doi: 10.1128/mBio.00837-19 (PMC6550523; doi:10.1128/mBio.00837-19)
Supplement: TABLE S4 [file mBio.00837-19-st004.pdf]

Supplementary Table 4.

Whole genome sequencing results of six chromosomal mutants selected on 6 mg/L kanamycin (DA54853, DA54847, DA54850) or amikacin (DA54862, DA54869, DA54871). Insertions of IS5 (*insH)*, IS1 (*insB)* and adenine (A) occurred upstream (us) of the corresponding gene.

**DA-number**	**gene**	**substitution**	**insertion/deletion**
DA54853	*cydA*		152us:IS5
DA54847	*abrB*	T293A	
	cydA	G266T	
	yffS		36us:A
DA54850	*potD*	A445T	
	mdtB	G1088A	
	ubiF		31_45del
DA54862	*ubiJ*		G270del
DA54869	*ubiF*		31_45del
DA54871	*cydA*		175us:IS1
