# Supplementary material for: De Novo Emergence of Peptides That Confer Antibiotic Resistance
Source: mBio. 2019 Jun 4;10(3):e00837-19. doi: 10.1128/mBio.00837-19 (PMC6550523; doi:10.1128/mBio.00837-19)
Supplement: TABLE S5 [file mBio.00837-19-st005.pdf]

Supplementary Table 5. List of transmembrane helices tested for increased aminoglycoside resistance.

Gene	Helix #	Sequence	Length (aa)	Increasing resistance
envZ	1	MLLLIVTLLFASLVTTYLVVL	21	No
	2	MPLFRYTLAIMLLAIGGAWLFI	22	No
phoQ	1	MFLLATAAVVLVLSLAYGMVAL	22	No
	2	MFIYVLSANLLLVIPLLWVAAW	22	No
pstC	1	MLAALIVLLMLGGIIVSLIIS	21	No
	2	MIYGTLVTSFIALLIAVPVSF	21	No
murP	1	MLIPGFIAAGLLLGIATLIATV	22	No
	2	MALNFMKVFSKGLFTFLVILVG	22	No
pitA	1	MFAGLDLHTGLLLLLALAFVLF	22	No
	2	MLAVVMAAVFNFLGVLLGGLSV	22	No
